# Afferent Projections to Area Prostriata of the Mouse

**DOI:** 10.3389/fnana.2020.605021

**Published:** 2020-11-27

**Authors:** Jin-Meng Hu, Chang-Hui Chen, Sheng-Qiang Chen, Song-Lin Ding

**Affiliations:** ^1^Key Laboratory of Neuroscience, School of Basic Medical Sciences, Institute of Neuroscience, The Second Affiliated Hospital, Guangzhou Medical University, Guangzhou, China; ^2^Allen Institute for Brain Science, Seattle, WA, United States

**Keywords:** visual cortex, retrosplenial cortex, auditory area, entorhinal cortex, claustrum, connecticity, cre-dependent tracing, anterior thalamic nuclei

## Abstract

Area prostriata plays important roles in fast detection and analysis of peripheral visual information. It remains unclear whether the prostriata directly receives and integrates information from other modalities. To gain insight into this issue, we investigated brain-wide afferent projections to mouse prostriata. We find convergent projections to layer 1 of the prostriata from primary and association visual and auditory cortices; retrosplenial, lateral entorhinal, and anterior cingulate cortices; subiculum; presubiculum; and anterior thalamic nuclei. Innervation of layers 2–3 of the prostriata mainly originates from the presubiculum (including postsubiculum) and anterior midline thalamic region. Layer 5 of the prostriata mainly receives its inputs from medial entorhinal, granular retrosplenial, and medial orbitofrontal cortices and anteromedial thalamic nucleus while layer 6 gets its major inputs from ectorhinal, postrhinal, and agranular retrosplenial cortices. The claustrum, locus coeruleus, and basal forebrain provide relatively diffuse innervation to the prostriata. Moreover, Cre-dependent tracing in cortical areas reveals that the cells of origin of the prostriata inputs are located in layers 2–4 and 5 of the neocortical areas, layers 2 and 5 of the medial entorhinal cortex, and layer 5 of the retrosplenial cortex. These results indicate that the prostriata is a unique region where primary and association visual and auditory inputs directly integrate with many limbic inputs.

## Introduction

Area prostriata (Pro) belongs to the limbic cortex, which is characterized by lack of granular layer 4 and existence of lamina dissecans (e.g., Sanides, 1969; Ding et al., 2003; Rockland, 2012). The Pro was described in primates including human brains about 50 years ago (Sanides, 1969; Allman and Kaas, 1971; Sousa et al., 1991; Morecraft et al., 2000), but the rodent equivalent of the primate Pro was not identified until recently (Ding, 2013; Lu et al., 2020). The Pro in both monkey and human brains was found to play important roles in fast analysis of information derived from the peripheral visual field (Yu et al., 2012; Mikellidou et al., 2017; Tamietto and Leopold, 2018). These functions are consistent with our recent finding that the rodent Pro receives strong afferent projections directly from the medial primary visual cortex (V1) which represents the peripheral visual field (Lu et al., 2020). Thus, the direct projections from V1 to the Pro could serve as the neural substrate for the fast analysis of peripheral visual information (Lu et al., 2020). Due to lack of sufficient information about Pro afferents, it is still not clear if the Pro integrates information from other sensory modalities such as auditory, somatosensory, and olfactory ones. In literature, the Pro in rodent was found to receive direct inputs from the subiculum (Sub; Ding et al., 2020), presubiculum (PrS), visual cortex, and anterior thalamic nuclei (ATN; Ding, 2013; Lu et al., 2020). However, detailed information about these inputs such as laminar distribution and intensity remains to be examined. In addition, it is also not clear whether other cortical and subcortical regions provide inputs to the Pro. In this article, we aim to investigate brain-wide afferent projections to the Pro of the mouse with emphasis on laminar organization of the inputs from different sources. We find that mouse Pro mainly receives and processes information from primary and secondary/association (we use the term “association” in this study) visual and auditory cortices, from the regions critical for spatial processing and navigation such as Sub, PrS, ATN, and medial entorhinal (MEC) and retrosplenial (RS) cortices and from the structures important for attention such as claustrum (Cla).

## Materials and Methods

### Animals

Adult male C57BL/6 wild-type mice (WT, ~58 cases) and a variety of Cre-driver lines of both sexes (~189 cases) at postnatal day 56 were included in this study (see “Results” section). All experiments were performed in accordance with the Guide for the Care and Use of Laboratory Animals of the Research Ethics Committee. Aseptic techniques were adopted during the whole surgery which was carried out under deep anesthesia (with 5% isoflurane) to ensure less injury and uncomfortability to animals (for details see http://help.brain-map.org/display/mouseconnectivity/documentation and Harris et al., 2019).

### Tracing Experiments

Raw data from anterograde viral injections in mouse brains were downloaded from Allen Mouse Brain Connectivity^1^ (see Harris et al., 2019). The methods for obtaining these data are published on the website^2^. Briefly, a pan-neuronal AAV vector expressing EGFP under the human synapsin I promoter (AAV2/1.pSynI.EGFP.WPRE.bGH) was injected in target regions of WT mice based on the coordinates of the mouse brain atlas (Paxinos and Franklin, 2012). The AAV vector binding with receptors expressed on affect soma in injection site results in permanent transgene (EGFP) expression in affect neurons *via* self-replication and finally yields whole neuronal labeling including dendrites, axons, and axon terminals. In Cre driver mice, a Cre-dependent AAV vector (AAV2/1.pCAG.FLEX.EGFP.WPRE.bGH) was injected. The Cre-dependent AAV vector selectively infects the soma expressing Cre in Cre-driver lines and produces cell type-specific anterograde tracing. The viral tracers were delivered by iontophoresis (current 3 μA and 7-s on/7-s off-duty cycle) for 5 min. After 21 days, mice were intracardially perfused with 4% paraformaldehyde and then stored in PBS with 0.1% sodium azide. The survival time was determined based on a pilot study to achieve the best tracing results. For imaging, brains were placed in 4.5% oxidized agarose, transferred to a phosphate buffer solution, and placed in a grid-lined embedding mold for standardized orientation in an aligned coordinate space. Multiphoton image acquisition was accomplished by using the TissueCyte 1000 system (TissueVision, Cambridge, MA, USA).

### *In situ* Hybridization (ISH)

Raw ISH data were derived from Allen Mouse Brain Atlas^3^ (see Lein et al., 2007). The details for generating these data including probe synthesis, primer design, tissue preparation, condition of hybridization, image processing, and quality control are published online^4^. Briefly, after sectioning, ISH of stains was performed on slides using a semi-automated non-isotopic digoxigenin-labeled colorimetric platform. Riboprobes were labeled with either digoxigenin-UTP or dinitrophenyl-11-UTP (DNP; Perkin Elmer, Waltham, MA, USA). A DNP-labeled probe and a DIG-labeled probe were hybridized simultaneously. Tyramide signal amplification was performed for each probe individually, using either anti-DIGHRP with tyramidebiotin or anti-DNP-HRP with tyramide-DNP for amplification.

### Data Analysis and Image Capture

**Figure 1:**
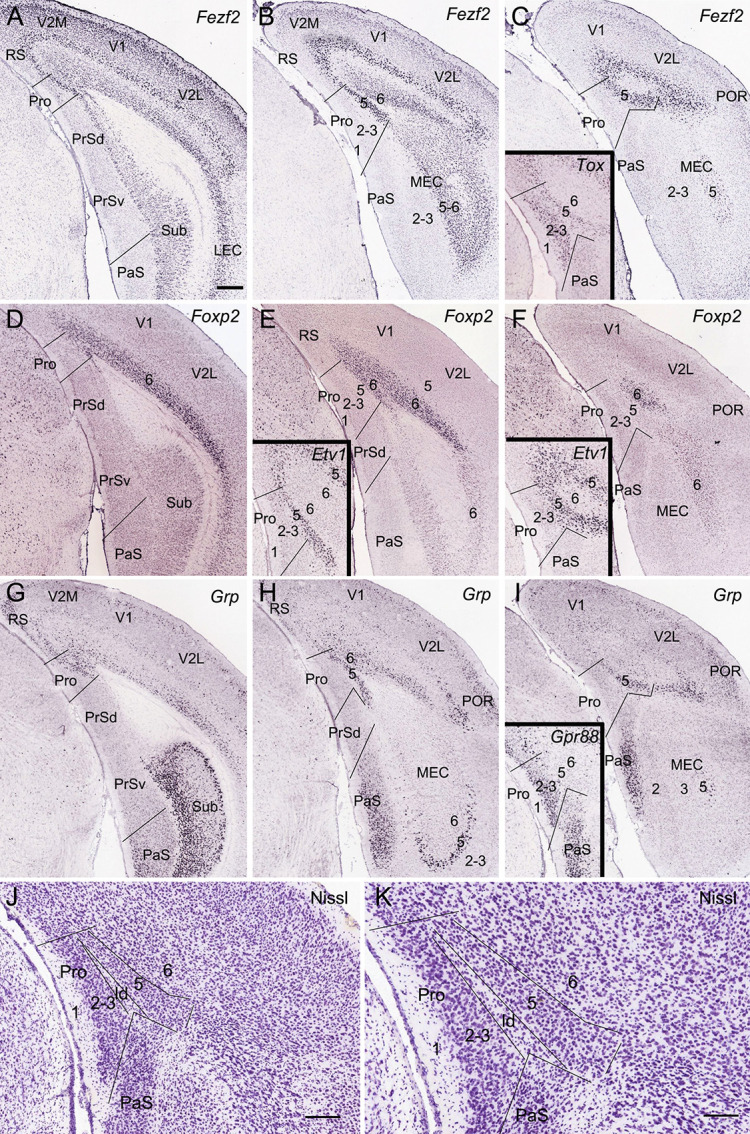
Location topography of mouse prostriata (Pro). Panels **(A–I)** and all the insets are ISH-stained sections, while panels **(J)** and **(K)** are Nissl-stained sections. Arabic numerals indicate cortical layers. **(A–C)** Three rostral **(A)** to caudal **(C)** coronal sections showing *Fezf2* expression in the Pro and adjoining regions. Strong *Fezf2* expression is mainly seen in layers 5 and 6 (L5–6) of the Pro, retrosplenial (RS), presubiculum (PrS), medial entorhinal cortex (MEC), and lateral entorhinal cortex (LEC) and neocortical regions (V2M, V1, V2L, and POR) as well as in the Sub. L2–3 of the visual cortices also show faint expression. The inset in panel **(C)** shows *Tox* expression in L2–3 and L5 of the Pro.** (D–F)** Three rostral **(D)** to caudal **(F)** sections showing *Foxp2* expression in L6 of the Pro and adjoining cortical regions. The insets in panels **(E,F)** show *Etv1* expression in L5 of the Pro and adjoining regions. **(G–I)** Three rostral **(G)** to caudal **(I)** sections showing *Grp* expression in the Pro and adjoining regions. Strong *Grp* expression is observed in L5–6 of the Pro, L2 of the RS, ventral part of the PaS, L5 of the MEC, and L2–3 and L5 of the postrhinal cortex (POR) as well as in the Sub. The inset in panel **(I)** shows *Gpr88* expression in the PaS and L2–3 of the Pro.** (J,K)** Cytoarchitecture and layers of the Pro shown at lower **(J)** and high **(K)** magnifications of a Nissl-stained section. Note the cell-sparse lamina dissecans (ld) separating L2–3 from L5 of the Pro. For abbreviations see the list. Bar: 350 μm in panel **(A)** (for **A–I** and all insets); 210 μm in panel **(J)**; 130 μm in panel **(K)**.

**Figure 2:**
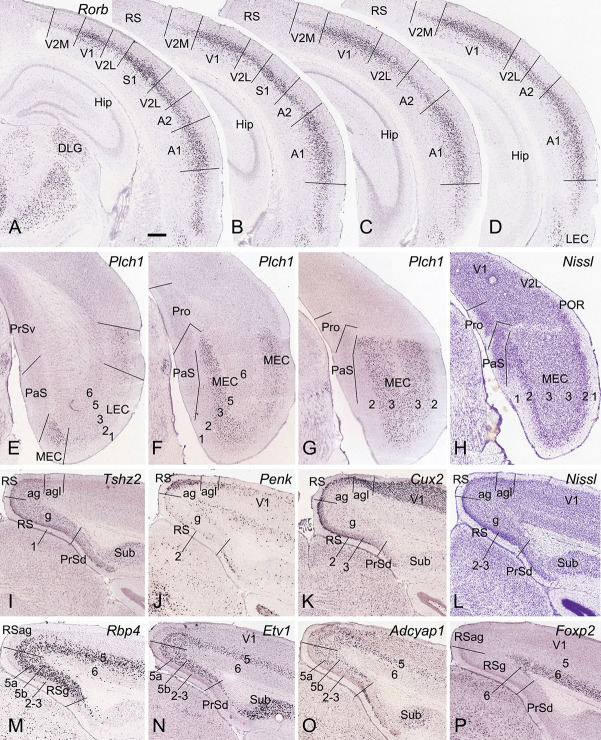
Locations and subdivisions of visual, auditory, entorhinal, and retrosplenial cortices. Panels **(A–G)** and **(I–K)** are ISH-stained sections while panels **(H,L)** are Nissl-stained sections. **(A–D)** Four rostral **(A)** to caudal **(D)** coronal sections showing *Rorb* expression in L4 of the primary and secondary visual and auditory cortices (V1, V2M, V2L, A1, and A2) as well as in the caudal S1. Note the strong and moderate *Rorb* expression in the primary (V1, S1, and A1) and secondary/association (A2, V2M, V2L) sensory cortices, respectively. No or little *Rorb* expression is seen in the RS. (**E–G)** Three rostral **(E)** to caudal **(G)** sections showing little and strong *Plch1* expression in L3 of the lateral and medial entorhinal cortex (LEC and MEC), respectively. **(H)** A Nissl-stained section showing the locations of the Pro, PaS, and L2 of the MEC. (**I–L)** Four sections stained respectively for *Tshz2* **(I)**, *Penk* **(J)**, *Cux2* **(K)**, and Nissl **(L)** showing the locations of three RS subdivisions (RSg, RSag, and RSagl), which display differential molecular signature and cytoarchitecture. **(M–P)** Four sections stained respectively for *Rbp4***(M)**, *Etv1* **(N)**, *Adcyap1* **(O)**, and *Foxp2* **(P)** showing lamination of the RS. Note the thick L5 in the RS, which can be subdivided into 5a and 5b. Bar: 350 μm in panel **(A)** (for all panels).

From the Allen Mouse Brain Connectivity Atlas portal, we first screened the tracing experiments which showed projections to the Pro using the Source Search tool (e.g., choose “gray” for whole brain search) and/or Spatial Search tool (e.g., choose area prostriata for its afferents). We then manually examined each selected tracing experiment to confirm the existence of labeled axon terminals and their laminar distribution in the Pro and to determine the precise injection sites and extents. The injection sites were determined by registering individual experiment brains to the Allen Mouse Brain Common Coordinate Framework (version 3; CCFv3) where all brain regions were comprehensively annotated (Harris et al., 2019; Wang et al., 2020), and with reference to the mouse brain atlas (Paxinos and Franklin, 2012) and other Nissl- and ISH-data (e.g., Figures 1, 2). Those cases with no terminal labeling in the Pro (except for somatosensory cortex, which was included as comparison with visual and auditory cortices) and those with contaminated injection sites were excluded from further analysis. Images from the regions of interest were selected. All selected images were imported into Adobe Photoshop for adjustment of brightness and contrast as well as arrangement and anatomical annotation.

**Figure 3:**
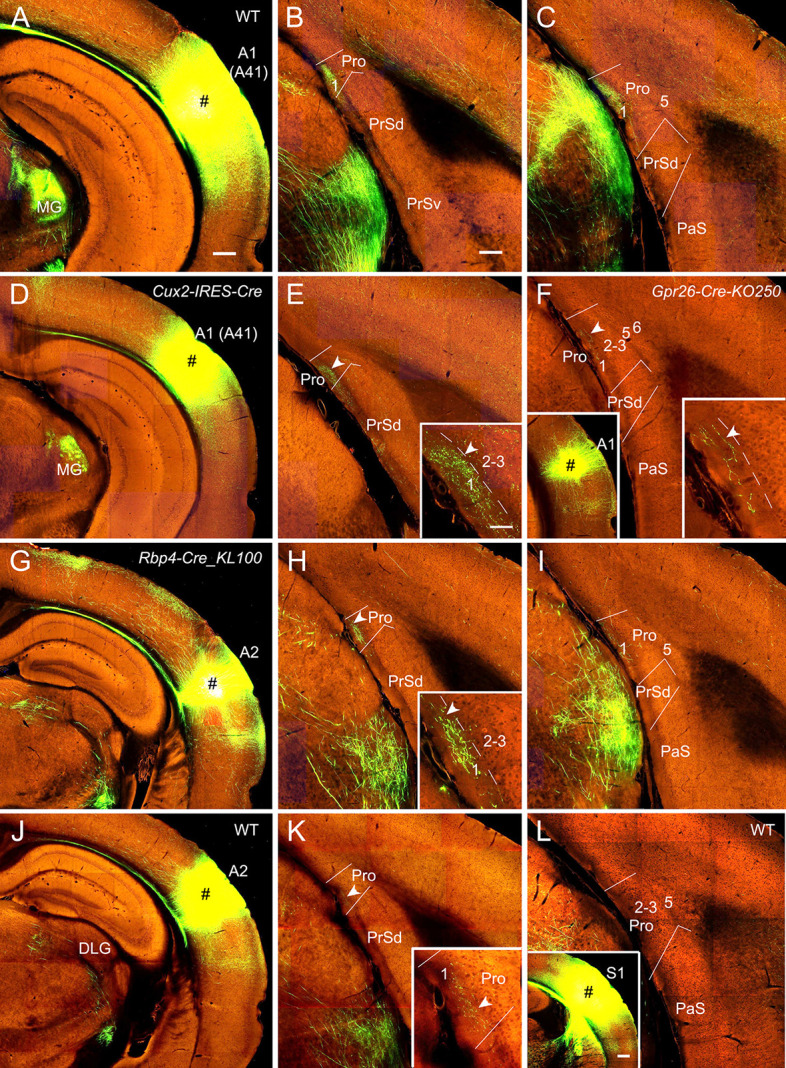
Projections from the auditory cortex to the Pro. For most cases, two coronal sections (one from rostral and the other from caudal levels) of the Pro are shown. **(A–C)** An injection in **A1** (i.e., Brodmann area 41; # in **A**) of a WT (wild type) mouse results in strong axon terminal labeling in L1 of the Pro **(B,C)**. **(D,E)** An injection in A1 (# in **D**) of a *Cux2*-IRES-Cre mouse gives rise to moderate terminal labeling in L1 of the Pro **(E)** with magnified image in the inset. **(F)** A small injection in A1 (# in the left inset) of a *Gpr26*-Cre_KO250 mouse produces weak terminal labeling in L1 of the Pro with magnified image in the right inset.** (G–I)** An injection in A2 (# in **G**) of a *Rbp4*-Cre_KL100 mouse yields moderate or weaker terminal labeling in L1 of the Pro **(H,I)**. The labeling in **(H)** is magnified in the inset.** (J,K)** An injection in A2 (# in **J**) of a WT mouse leads to weak terminal labeling in L1 of the Pro **(K)**, which is magnified in the inset. **(L)** An injection in S1 (# in the inset) of a WT mouse leads to no terminal labeling in the Pro. Arrowheads in low and high magnification images of the Pro point to corresponding locations. Bars: 350 μm in panel **(A)** (for **A**, **D**, **G**, **J** and the left inset in **F**); 200 μm in **(B)** (for other panels); 350 μm in the inset in **(L)**; and 70 μm in the inset in **(E)** (for all other insets).

The intensity of labeled axon terminals in the Pro was semi-quantified. “Strong” labeling indicates that the terminals are very densely or densely distributed and can be easily and clearly observed even at low magnification (e.g., Figures 3B,C). “Moderate” labeling means that the terminals are less densely distributed and cannot be clearly seen at low magnification and thus need to be confirmed at higher magnification (e.g., Figures 3E,H). “Weak” labeling means loosely or very loosely distributed terminals that are barely visible at low magnification (e.g., Figures 3F,K). When variable grading occurs along the rostral–caudal axis of the Pro, we use highest grading for the Pro.

### Nomenclature

Most of the terminology used in this study is based on the commonly used mouse brain atlases: the Allen Mouse Brain CCFv3 (Wang et al., 2020) which has the Pro annotated and the Paxinos’s atlas which does not have Pro annotation (Paxinos and Franklin, 2012). In both atlases, the full names of most structures are identical or similar but with different abbreviations. For details, see the list of abbreviations for the structures described in this study. Since it is difficult to identify specific subregions in the association visual and auditory cortices, we use V2 (V2M for medial and V2L for lateral parts) and A2 to group these two, respectively. Additionally, we treat the postsubiculum as dorsal PrS (PrSd; also see Paxinos and Franklin, 2012) and the original PrS as ventral PrS (PrSv). The RS is subdivided into granular (RSg), agranular (RSag), and lateral agranular (RSagl) parts (Wang et al., 2020) with each displaying different molecular and cellular features (see “Results” section below).

## Results

### Identification of the Pro and Related Cortical Regions

As detailed recently (Lu et al., 2020), the Pro is located at the junction of the PrS, parasubiculum (PaS), RS, and the medial visual cortices (V2M and V1; Figures 1A–I). Compared to its neighbor PrSd, the Pro has unique layers 2–3 (L2–3) which are difficult to separate from each other, a thicker L5 and a thinner L6 (Figures 1J,K). Both L2–3 and L5 have their specific molecular markers such as *Calb1* and *C1ql2*, respectively (see Lu et al., 2020). Other molecular markers can also be used for identifying the lamination of the Pro. To further confirm the definition of L2–3, L5, and L6 of the Pro, six molecular markers were used in this study. Fez family zinc finger 2 (*Fezf2*) is strongly and continuously expressed in L5 of the Pro and of all adjoining cortical regions including PrSd, PrSv, RS, V2M, V1, V2L, and MEC (Figures 1A–C).* Fezf2* is also expressed in L6 of these cortical regions and in the Sub. *Tox* (thymocyte selection-associated high-mobility group box) is expressed in L2–3 and L5 of the Pro (inset in Figure 1C). *Foxp2* (forkhead box P2) displays expression in L6 of the Pro, RS, and neocortical areas (Figures 1D–F) while ets variant 1 (*Etv1*) shows expression in L5 of the Pro and other cortical regions (insets in Figures 1E,F). gastrin-releasing peptide (*Grp*) displays differential expression patterns in different cortical regions. Specifically, *Grp* expresses strongly in Sub, L5, and L6 of the Pro, L2, and L5–6 of the RS, L2–3, L5, and L6 of the postrhinal cortex (POR), L2–3 of the PaS and L5 of the MEC with moderate expression in L2, L5, and L6 of the V2M and V1 (Figures 1G–I). G-protein coupled receptor 88 (*Gpr88*) is expressed in the PaS and L2–3 of the Pro (inset in Figure 1I).

To identify the primary somatosensory cortex (S1), primary auditory cortex (A1), and V1, we used the gene marker RAR-related orphan receptor beta (*Rorb*). *Rorb* is strongly expressed in L4 of the S1, A1, and V1 and moderately expressed in L4 of adjoining A2, V2, and ectorhinal cortex (ECT; Figures 2A–D). In the lateral entorhinal cortex (LEC), *Rorb* expression is observed mainly in deep layers (Figure 2D). In the MEC, phospholipase C, eta 1 (*Plch1*) is selectively expressed in L3 with few in LEC (Figures 2E–G). L2 of the MEC can be easily identified in Nissl-stained sections because of its large cells (e.g., Figure 2H). Identification of RSg, RSag, and RSagl of the RS is based on regional expression of some genes such as teashirt zinc finger family member 2 (*Tshz2*), preproenkephalin (*Penk*), and cut-like homeobox 2 (*Cux2*). *Tshz2* is expressed in L2–3 and L5 of RSg and RSag but not RSagl (Figure 2I).* Penk* is strongly expressed in L2–3 of RSag with much less in RSg and RSagl (Figure 2J). *Cux2* displays strong expression in L2–3 of RSg and RSagl with less in RSag (Figure 2K). On Nissl-stained sections, RSg can also be easily separated from RSag and PrSd because of its densely packed granular L2–3 (e.g., Figure 2L). Laminar identification of the RS is based on some layer-selective gene markers. For example, *Cux2* is expressed in L2–3 (Figure 2K); *Rbp4*, *Etv1*, and *Adcyap1* in L5 (Figures 2M–O), and *Foxp2* in L6 (Figure 2P). Taken together, with region- and layer-selective molecular markers the Pro and adjoining regions (e.g., PrS, PaS, MEC, and RS) can be reliably identified on traditional coronal sections.

### Projections From Primary and Association Auditory and Somatosensory Cortices

In WT mice (two cases), injections of anterograde viral tracers into A1 (i.e., Brodmann area 41; e.g., Figure 3A) result in strong terminal labeling in L1 of the Pro with no or little in other layers (e.g., Figures 3B,C). As expected, the medial geniculate nucleus (MG) contains dense terminal labeling (Figure 3A) originated from A1 (mostly from L6). Results from A1 injections in *Emx1*-IRES-Cre mice (two cases; Cre expression in excitatory neurons) are similar to those from WT mice (not shown). To determine laminar origins of these projections to the Pro, we examined some layer-selective Cre-driver mice with injections mainly involved in A1. Terminal labeling is found in L1 of the Pro in *Cux2*-IRES-Cre (three cases; Cre expression in L2–4; e.g., Figures 3D,E; moderate labeling), *Rorb*-IRES2-Cre (one case) and *Scnn1a*-Tg3-Cre (one case; Cre in L4; not shown), *Rbp4*-Cre_KL100 (one case), and *Gpr26*-Cre_KO250 (one case; Cre in L5; e.g., Figure 3F; weak labeling) mice but not in *Rasgrf2*-T2A-dCre (one case; Cre in L2–3), *Ntsr1*-Cre_GN220 (2 cases; Cre in L6), and *Syt6*-Cre_KI148 (one case; Cre in L6) mice. These results together suggest that subsets of L4 and L5 but L6 neurons mainly originate A1 projections to the Pro although cells of origin from other subsets of L2–3 neurons cannot be totally ruled out.

Similarly, after tracer injections in the auditory association cortex (A2) of some Cre-driver lines and WT mice, consistent but weaker terminal labeling is observed in L1 of the Pro, for example, in *Cux2*-IRES-Cre (three cases; not shown), *Rbp4*-Cre_KL100 (one case; Figures 3G–I), *Tlx3*-Cre_PL56 (one case; not shown), *Emx1*-IRES-Cre (two cases; not shown), and WT (one case; Figures 3J,K) mice but not in *Ntsr1*-Cre_GN220 mice (three cases; not shown). No terminal labeling was detected in the Pro of *Etv1*-CreERT2 (one case; Cre in L5) and *Npr3*-IRES2-Cre (one case; Cre in L5) mice (not shown). In addition, injections in A1 and A2 of the Cre-driver lines for interneurons (e.g., *Gad2*-IRES-Cre, *Pvalb*-IRES-Cre, *Vip*-IRES-Cre, and *Sst*-IRES-Cre lines) produced no terminal labeling in the Pro, suggesting that interneurons in the auditory cortex does not project to the Pro.

In contrast to the auditory cortex, injections in the primary (S1) and secondary somatosensory cortices (30 different WT and Cre-driver cases) yield no terminal labeling in the Pro (e.g., Figure 3L).

### Projections From Association Visual Cortices

**Figure 4:**
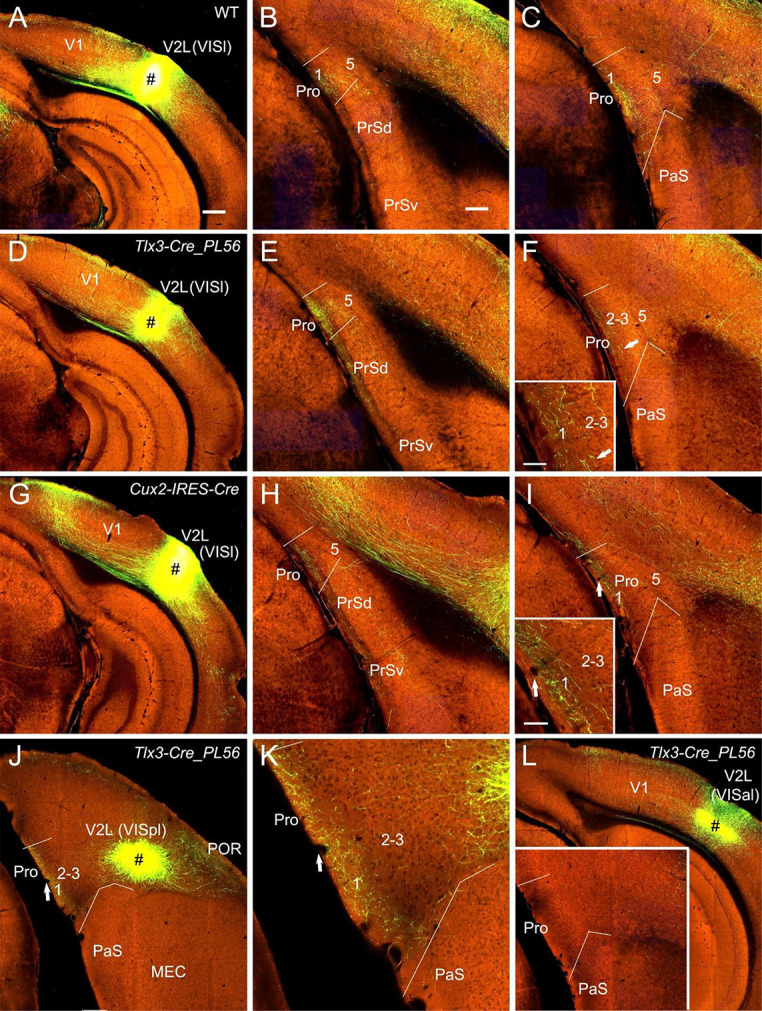
Projections from lateral visual cortices to the Pro.** (A–C)** An injection in the V2L-VISl subfield (# in **A**) of a WT mouse results in strong and weak terminal labeling in L1 and L2–3 of the Pro, respectively **(B,C)**. (**D–F)** An injection in the V2L-VISl subfield (# in **D**) of a *Tlx3*-Cre_PL56 mouse (Cre expression in L5) leads to strong and weak terminal labeling in L1 and L2–3 of the Pro, respectively **(E,F)**. The Pro labeling in **(F)** is magnified in the inset.** (G–I)** An injection in the V2L-VISl subfield (# in **G**) of a *Cux2*-IRES-Cre mouse (Cre in L2–4) produces moderate terminal labeling in L1 of the Pro **(H,I)**. The Pro labeling in **(I)** is magnified in the inset.** (J,K)** An injection in V2L-VISpl subfield (# in **K**) of a *Tlx3*-Cre_PL56 mouse results in moderate terminal labeling in L1 of the Pro **(K)**. The Pro labeling in **(J)** is magnified in **(K)**.** (L)** An injection in the VISal subfield of V2 (#) of a *Tlx3*-Cre_PL56 mouse does not result in terminal labeling in the Pro (see the inset). Arrows in low- and high-magnification images of the Pro point to corresponding locations. Bars: 350 μm in **(A)** (for **A**, **D**, **G**, **J**, and **K**); 200 μm in **(B)** (for other panels and the inset in **J**); 70 μm in the inset in **(F)** and **(I)**.

Since strong and weak projections has recently been described from V1 and V2M to the Pro (L1–3), respectively (Lu et al., 2020), here we focus on the projections from association visual cortices [mainly V2L, POR, and ECT (i.e., Brodmann area 36)] to the Pro. V2L mainly consists of subfields VISl, VISpl, and VISal (Wang et al., 2020). To examine whether V2L injections lead to various densities of labeled axon terminals in Pro, a lot of data from both WT and Cre-driver mice were analyzed. We find that VISl injections result in moderate to strong terminal labeling in the Pro (mainly in L1 with much less in L2–3 and no in L5–6) of WT (10 cases; e.g., Figures 4A–C), *Emx1*-IRES-Cre (13 cases; not shown), *Tlx3*-Cre_PL56 (22 cases; e.g., Figures 4D–F), and *Cux2*-IRES-Cre (18 cases; Figures 4G–I) mice. Similarly, VISpl injections lead to moderate terminal labeling in L1 of the Pro (2* Rbp4*-Cre_KL100 and 2* Tlx3*-Cre_PL56 mice) with an example from a *Tlx3*-Cre_PL56 mouse shown in Figures 4J,K. However, VISal injections produce no terminal labeling in the Pro (one WT, 2 *Rbp4*-Cre_KL100 and 3 *Tlx3*-Cre_PL56 mice; e.g., Figure 4L). No labeling was seen in the Pro of all V2L cases with injections in *Ntsr1*-Cre_GN220 and *Syt6*-Cre_KI148 mice (five cases each; not shown).

**Figure 5:**
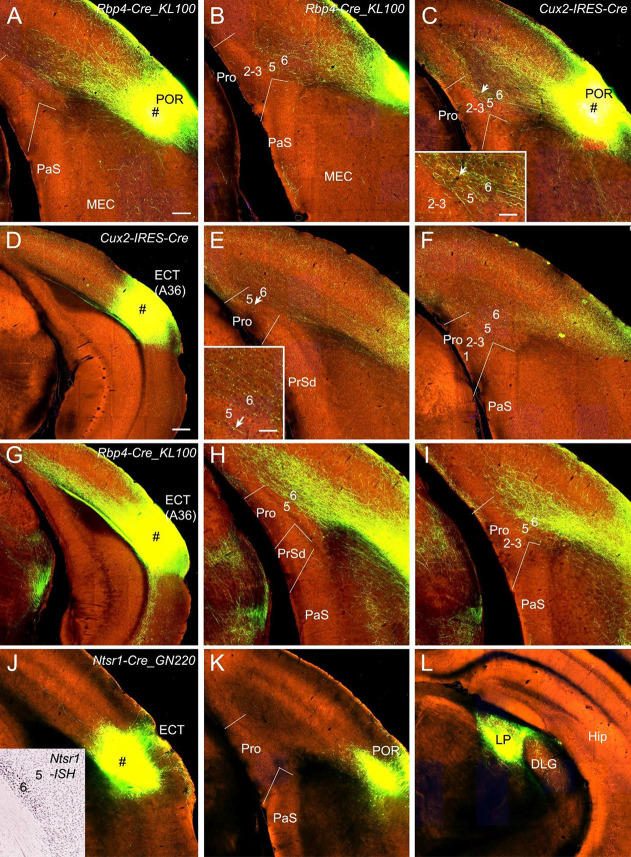
Projections from postrhinal and ectorhinal cortices to the Pro. **(A,B)** An injection in POR (# in **A**) of a *Rbp4*-Cre_KL100 mouse (Cre expression in L5) produces moderate and weak terminal labeling in L6 and L5 of the Pro, respectively **(A,B)**. **(C)** An injection in POR (#) of a *Cux2*-IRES-Cre mouse (Cre in L2–4) leads to moderate terminal labeling in L6 (with less in L5) of the Pro, which is magnified in the inset. (**D–F**) An injection in ECT (i.e., Brodmann area 36; # in **D**) of a *Cux2*-IRES-Cre mouse (Cre in L2–4) results in weak terminal labeling in L6 of the Pro **(E,F)**. The labeling in L6 of the Pro is magnified in the inset. (**G–I**) An injection in ECT (# in **G**) of a *Rbp4*-Cre_KL100 mouse (Cre in L5) leads to strong terminal labeling in L6 of the Pro **(H,I)**.** (J–L)** An injection in ECT (# in **J**) of a *Ntsr1-*Cre_GN220 mouse (Cre in L6; see the inset in **J** for ISH) produces no labeling in the Pro **(K)** and strong labeling in lateroposterior nucleus (LP in **L**). Arrows in low- and high-magnification images of the Pro point to corresponding locations. Bars: 200 μm in **(A)** (for **A–C**, **E, F, H, I, J–L**, and the inset in **J**); 350 μm in **(D)** (for **D,G**); 70 μm in the insets in **(C)** and **(E)**.

POR and ECT injections yield different projection patterns. Injections in the POR result in moderate terminal labeling in L6 and weak labeling in L5 of the Pro in *Rbp4*-Cre_KL100 (one case; Figures 5A,B) and *Cux2*-IRES-Cre (one case; Figure 5C) mice. ECT injections lead to weak and strong terminal labeling in L6 of the Pro in *Cux2*-IRES-Cre (one case; Figures 5D–F) and *Rbp4*-Cre_KL100 (one case; Figures 5G–I) mice, respectively. Injections in the ECT of *Ntsr1*-Cre_GN220 mice (two cases) produce no labeling in the Pro (e.g., Figures 5J,K) although strong terminal labeling is seen in the lateroposterior nucleus–pulvinar complex (LP; Figure 5L).

### Projections From Entorhinal and Piriform Cortices

**Figure 6:**
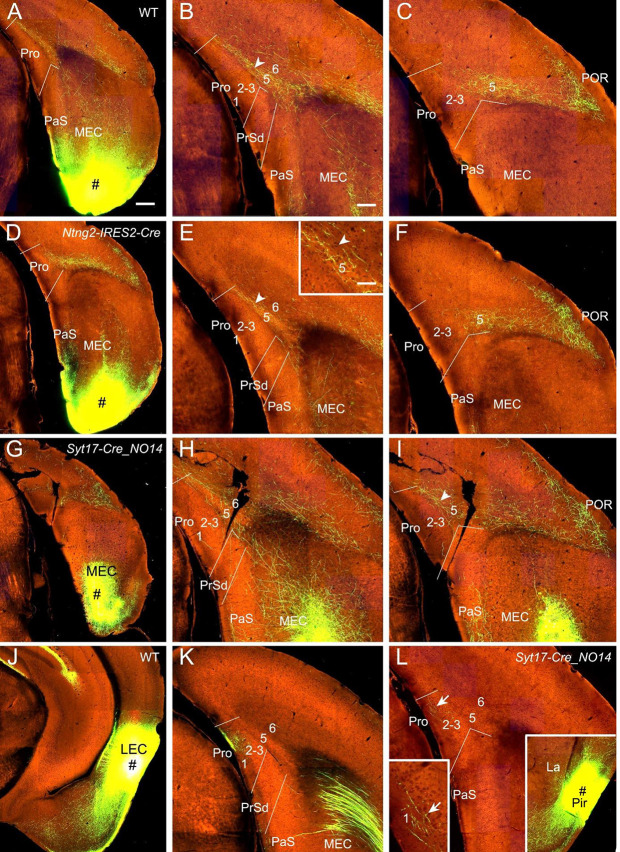
Projections from entorhinal and piriform cortices to the Pro.** (A–C)** An injection in MEC (# in **A**) of a WT mouse yields strong terminal labeling in L5 of the Pro **(B,C)**. (**D–F)** An injection in MEC (# in **D**) of a *Ntng2*-IRES2-Cre mouse (Cre expression in L2) produces moderate terminal labeling in L5 of the Pro **(E,F)**. The Pro labeling in **(E)** is magnified in the inset. (**G–I)** An injection in MEC (# in **G**) of a *Syt17*-Cre_NO14 mouse (Cre in L2 and L5) results in moderate terminal labeling in L5 of the Pro **(H,I)**. **(J,K)** One LEC injection (# in **J**) in a WT mouse results in strong and weaker terminal labeling in L1 and L2–3 of the Pro, respectively **(K)**. **(L)** Weak terminal labeling in L1 of the Pro after an injection was placed in the piriform cortex (# in the right inset) of a* Syt17*-Cre_NO14 mouse (Cre in L2–3). The labeling in L1 of the Pro is magnified in the left inset. Arrowheads and arrows point to the labeling in L5 and L1, respectively. Bars: 350 μm in **(A)** (for **A**, **D**, **G**, **J**, and the right inset in **L**); 200 μm in **(B)** (for other panels); 70 μm in the inset in **(E)** (also for the left inset in **L**).

Viral tracer injections in the MEC of WT (two cases; e.g., Figure 6A), *Ntng2*-IRES2-Cre (one case; Cre in L2; Figure 6D), *Rbp4*-Cre_KL100 (two cases; Cre in L5; not shown), *Grp*-Cre_KH288 (one case; Cre in L5; not shown), and *Syt17*-Cre_NO14 (one case; *Syt17*-Cre in L2 and L5; Figure 6G) mice lead to moderate to strong terminal labeling in L5 of the Pro with less in L6 and no in other layers (Figures 6B,C,E,F,H,I). Since *Ntng2*-Cre and *Rbp4*-Cre/*Grp*-Cre are respectively expressed in L2 and L5 of the MEC, these results suggest that subsets of neurons in L2 and L5 of the MEC are the major origins of the projections to the Pro.

In contrast to MEC, injections in the LEC of *Cux2*-IRES-Cre (two cases; Cre in L2–3) and WT (five cases) mice result in strong terminal labeling in L1 with less in L2–3 and no in L5–6 (e.g., Figures 6J,K). Data related to Cre-driver lines for L6 neurons of the MEC and LEC are not available (*Ntsr1* and *Syt6* are not expressed in L6 of the EC).

In 20 different WT and Cre-driver mice with injections in the piriform cortex (Pir) weak terminal labeling in L1–3 of the Pro was detected in three cases [two WT and one *Syt17*-Cre_NO14 (Cre in L2–3) mice]. The result from the *Syt17*-Cre mouse is shown in Figure 6L.

### Projections From Frontal and Retrosplenial Cortices

**Figure 7:**
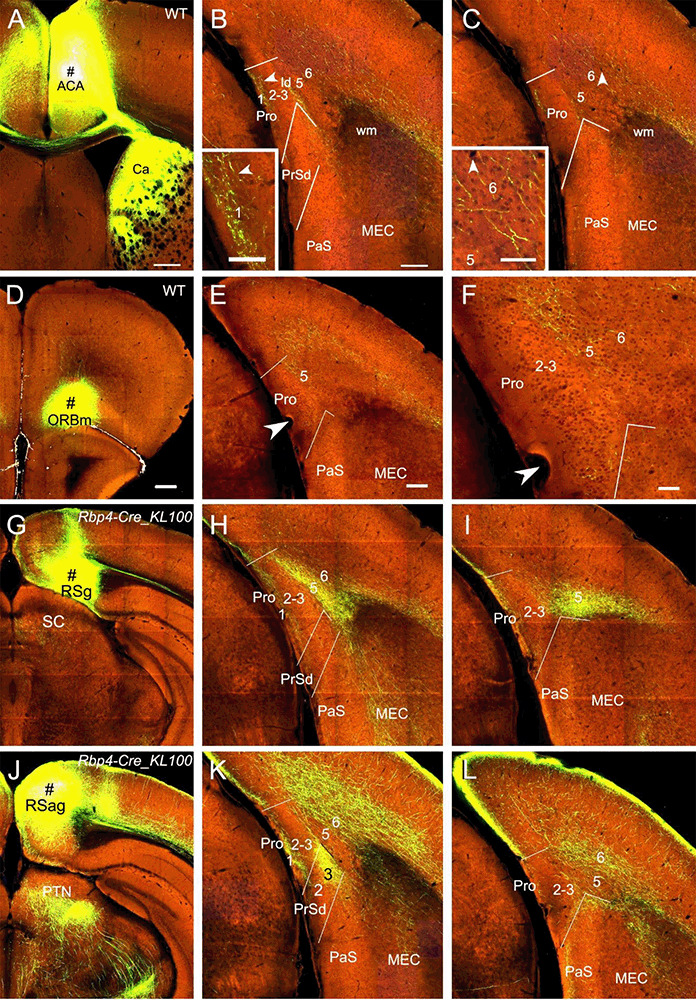
Projections from frontal and retrosplenial cortices to the Pro.** (A–C)**. An injection in the medial frontal cortex (i.e., anterior cingulate area, ACA; # in **A**) of WT mouse produces moderate and weak terminal labeling in L1 and L6 of the Pro, respectively **(B,C)**. Lamina dissecans (ld) also contains moderate labeling. The labeling in L1 and L6 of the Pro is magnified in the insets in **(B)** and **(C)**, respectively. Note the very strong terminal labeling in the dorsal caudate (Ca in **A**).** (D–F)** An injection in the medial orbitofrontal cortex (ORBm, i.e., Brodmann area 11; # in **D**) of a WT mouse gives rise to moderate terminal labeling in L5 of the Pro **(E,F)**. The Pro labeling in **(E)** is magnified in **(F)**. **(G–I)** An injection in RSg (# in **G**) of a *Rbp4*-Cre_KL100 mouse (Cre expression in L5) results in moderate and strong terminal labeling in L1 and L5 of the Pro, respectively **(H,I)**.** (J–L)** An injection in RSag (# in **J**) of a *Rbp4*-Cre_KL100 mouse leads to moderate and strong terminal labeling in L1 and L6 of the Pro, respectively **(K,L)**. Note that RSag but not RSg projects heavily to L3 of the PrSd **(K)**. Arrowheads in low- and high-magnification images of the Pro point to corresponding locations. Bars: 350 μm in **(A)**; 200 μm in **(B)** (also for **C**); 70 μm in the insets in **(B)** and **(C)**; 350 μm in **(D)** (for **D**, **G**, and **J**); 200 μm in **(E)** (for other panels).

Injections in the medial frontal cortex (i.e., anterior cingulate area, ACA; # in Figure 7A) of two WT mice lead to moderate and weak terminal labeling in L1 and L6 of the Pro, respectively (e.g., Figures 7B,C). The labeling in L6 but not L1 of the Pro was also found in 4 out of 28 Cre-driver lines: two *Rbp4*-Cre (Cre in L2, L3, and L5), one *Gpr26*-Cre_KO250 (Cre in L2, L3, and L5) and one *Grp*-Cre_KH288 (Cre in L2 and L5) mice (not shown). With injections in the medial orbitofrontal cortex (ORBm), weak and moderate terminal labeling is seen in L2–3 and L5 of the Pro, respectively, in 2 out of 25 cases [one Cux2-IRES-Cre (Cre in L2–3; not shown) and one WT (Figures 7D–F) mice].

Injections in RSg, RSag, and RSagl lead to strong terminal labeling in the Pro but with different laminar distribution in both WT (five cases; not shown) and Cre-driver mice (17 cases for RSg, 11 cases for RSag, and 12 cases for RSagl injections). Specifically, in WT and *Rbp4*-Cre_KL100 (10 cases) mice, RSg mostly projects to L1 and L5 (e.g., Figures 7G–I) while RSag and RSagl projects mostly to L1 and L6 (e.g., Figures 7J–L). Since *Rbp4*-Cre is expressed in L5 of the RS (see Figure 2M), these findings suggest that L5 of the RS originates most of the projections to the Pro. Axon terminal labeling in the Pro was also observed in *A930038C07Rik*-Tg1-Cre (five cases; Cre in L5) and *Drd3*-Cre-KI196 (three cases; Cre in L5) mice after RS injections (not shown). However, in other Cre-driver lines targeting L5 neurons such as *Etv1*-CreERT2 (one case), *Adcyap1–*2A-Cre (one case), and *Efr3a*-Cre_NO108 (two cases), RS injections did not result in terminal labeling in the Pro (not shown). In *Cux2*-IRES-Cre (five cases; Cre in L2–3), *Syt6*-Cre_KI148 (three cases; Cre in L6), and *Ntsr1*-Cre_GN220 (five cases; Cre in L6) mice, no labeling was found in the Pro (not shown), suggesting L6 of the RS do not project to the Pro.

### Projections From the Subicular Complex

**Figure 8:**
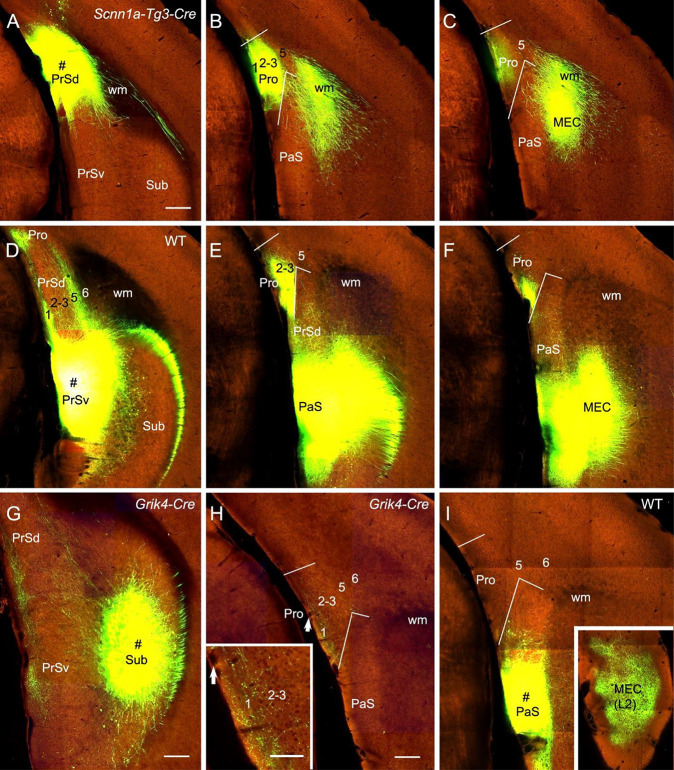
Projections from subicular cortices to the Pro. (**A–C)** An injection in the PrSd (i.e., PoS; # in **A**) of one *Scnn1a*-Tg3-Cre mouse (Cre expression in L2–3) results in strong terminal labeling in L1–3 of the Pro and the MEC **(B,C)**. (**D–F)** An injection in the PrSv (# in **D**) of one WT mouse leads to strong terminal labeling in L1–3 of the Pro and the MEC **(E,F)**. Note that the PrSv axons reach the Pro through L1 of the PrS and reach the MEC through the PaS. In contrast, PrSd axons reach the MEC *via* the white matter (wm).** (G,H)** An injection in the Sub (# in **G**) of a *Grik4*-Cre mouse (Cre in Sub) leads to moderate terminal labeling in L1–3 of the Pro **(H)**. The Pro labeling in panel **(H)** is magnified in the inset. Arrows in the Pro point to corresponding locations.** (I)** An injection in the PaS (# in **I**) of WT mouse produces no terminal labeling in the Pro but strong labeling in L2 of the MEC (inset). Bars: 280 μm in **(A)** (for **A–F**); 350 μm in **(G)**; 200 μm in **(H)** (also for **(I)** and the inset in **I**); 100 μm in the inset in **(H)**.

The subicular complex includes prosubiculum, Sub, PrS and PaS (Ding, 2013). A recent study has shown that prosubiculum does not project to the Pro while Sub heavily projects to L1–3 of the Pro (Ding et al., 2020). In this study, we have mainly examined the projections from PrS and PaS to the Pro (eight cases). Both PrSd [one *Drd3*-Cre_KI196 (Cre expression in L2–3) and two *Scnn1a*-Tg3-Cre (Cre in L2–3) mice] and PrSv [one WT, one *Grm2*-Cre_MR90 (Cre in L2–3) and one *Slc17a6*-IRES-Cre (Cre in L2–3) mice] project strongly to L1–3 of the Pro and L2–3 of the MEC. Examples from one *Scnnla*-Tg3-Cre (Figures 8A–C) and one WT (Figures 8D–F) mice are shown. PaS injections [one WT and one *Wfs1*-Tg2-CreERT2 (Cre in L2–3) mice] lead to no and strong terminal labeling in the Pro and MEC, respectively (e.g., Figure 8I). PrSd projections appear to reach to the Pro *via* direct caudal extension without going through the white matter (wm; Figures 8A–C). In contrast, axons of PrSv projections to the Pro appear to go through L1 of the PrS before reaching to the Pro (Figures 8D–F). Additionally, PrSd projects to MEC *via* the wm in (Figures 8B,C) while PrSv projects caudally to MEC *via* the PaS without going through the wm (Figures 8E,F). Injections in the Sub (two WT and 10 different Cre-driver mice; see Ding et al., 2020) consistently yield moderate to strong terminal labeling in L1–3 of the Pro. An example from a Grik4-Cre (Cre in Sub) mouse is shown in Figures 8G,H.

### Projections From Subcortical Regions

**Figure 9:**
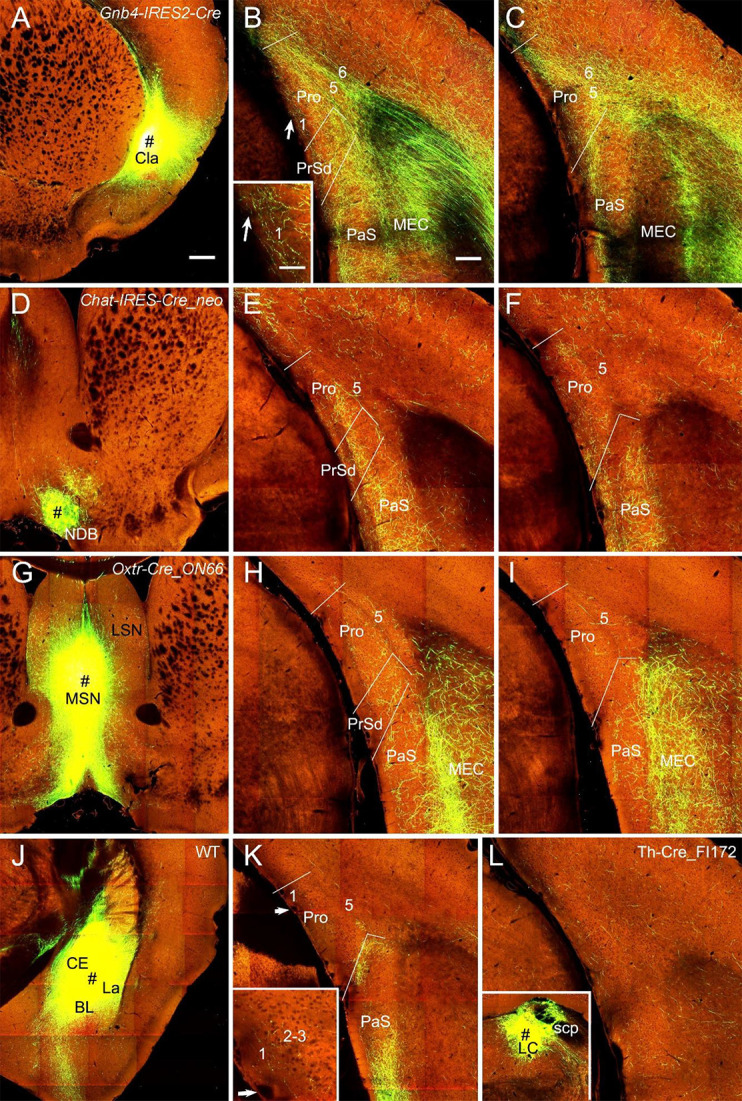
Projections from subcortical regions to the Pro. **(A–C)** An injection in the claustrum (Cla) of *Gnb4*-IRES-Cre mouse (Cre expression in Cla) results in obvious labeling in all layers of the Pro and adjoining regions. Moderate terminal labeling in L1–3 and strong labeling in L5–6 of the Pro is observed **(B,C)**. The labeling in L1 of the Pro in **(B)** is magnified in the inset. **(D–F)** An injection in the nucleus of diagonal band (NDB; # in **D**) of a *Chat*-IRES-Cre_neo mouse (Cre in NDB) yields terminal labeling in L1–3 of the Pro with less in L5–6 **(E,F)**. Stronger labeling is seen in PaS. **(G–I)** An injection in medial septal nucleus (MSN; # in **G**) of an *Oxtr*-Cre_ON66 mouse (Cre in MSN) gives rise to weak terminal labeling in L2–5 of the Pro **(H,I)**. Note the relatively stronger labeling in PrSd and PaS and the very strong labeling in the medial portion of the MEC. **(J,K)** An injection in the amygdalar region (CE, BL, and La; # in **J**) of a WT mouse results in weak terminal labeling in L1–3 of the Pro **(K)**. The Pro labeling in **(K)** is magnified in the inset. Injections restricted in the central nucleus (CE) or basolateral nucleus (BL) yields no labeling in the Pro (not shown). **(L)** Weak terminal labeling in L1–6 of the Pro after an injection was placed in locus coeruleus (LC) of a Th-Cre_FI172 mouse (# in the inset in **L**). Bars: 350 μm in **(A)** (for **A**, **D**, **G**, **J**, and inset in **L**); 200 μm in **(B)** (for other panels); 70 μm in the inset in **(B)** (also for the inset in **K**).

In both WT (one case) and Cre-driver (1 *Gnb4*-IRES2-Cre and 2 *Ntng2*-IRES-Cre) mice the claustrum (Cla) projects strongly and diffusely to the Pro. For example, the injection in the Cla of a *Gnb4*-IRES2-Cre mouse (Figure 9A) results in clear terminal labeling in all layers of the Pro as well as in adjoining regions such as PrS and PaS (Figures 9B,C). Moderate and strong terminal labeling is seen in L1–3 and L5–6 of the Pro, respectively (Figures 9B,C). Tracer injections in the nucleus of diagonal band (NDB) of two *Ntrk1*-IRES-Cre and two *Chat*-IRES-Cre-neo mice (e.g., Figure 9D) yield moderate labeling in L2–3, weak labeling in L1 and L5–6, and strong labeling in the PaS (e.g., Figures 9E,F). Injections in the lateral septal nucleus produced no labeling in the Pro (not shown) while those in the medial septal nucleus (MSN; 1 *Chat*-IRES-Cre-neo, 1 *Tacr1*-T1A-Cre, and 1 *Oxtr*-Cre_ON66 mice) revealed weak labeling in L2–3 and L5. The result from the *Oxtr*-Cre_ON66 mouse is shown in Figures 9G–I. Relatively stronger labeling in PrSd and PaS and very strong labeling in the medial portion of the MEC (mostly in L2–3) are also observed (Figures 9H,I). The projections from the amygdala to the Pro are rare. Scattered weak terminal labeling exists in L2–3 of the Pro in 2 out of 20 cases with injections involved in the lateral amygdala nucleus. An example from one WT mouse is shown in Figures 9J,K. No labeling in the Pro was observed in cases with injections in other amygdala nuclei including central and basolateral nuclei (not shown). The injection in the locus coeruleus [LC; one Th-Cre_FI172 (Cre in LC) mouse] results in weak terminal labeling in L1–6 of the Pro (Figure 9L). In addition, an injection involved in the lateroposterior nucleus–pulvinar complex of a WT mouse produced weak terminal labeling in L5–6 of the Pro (not shown).

**Figure 10:**
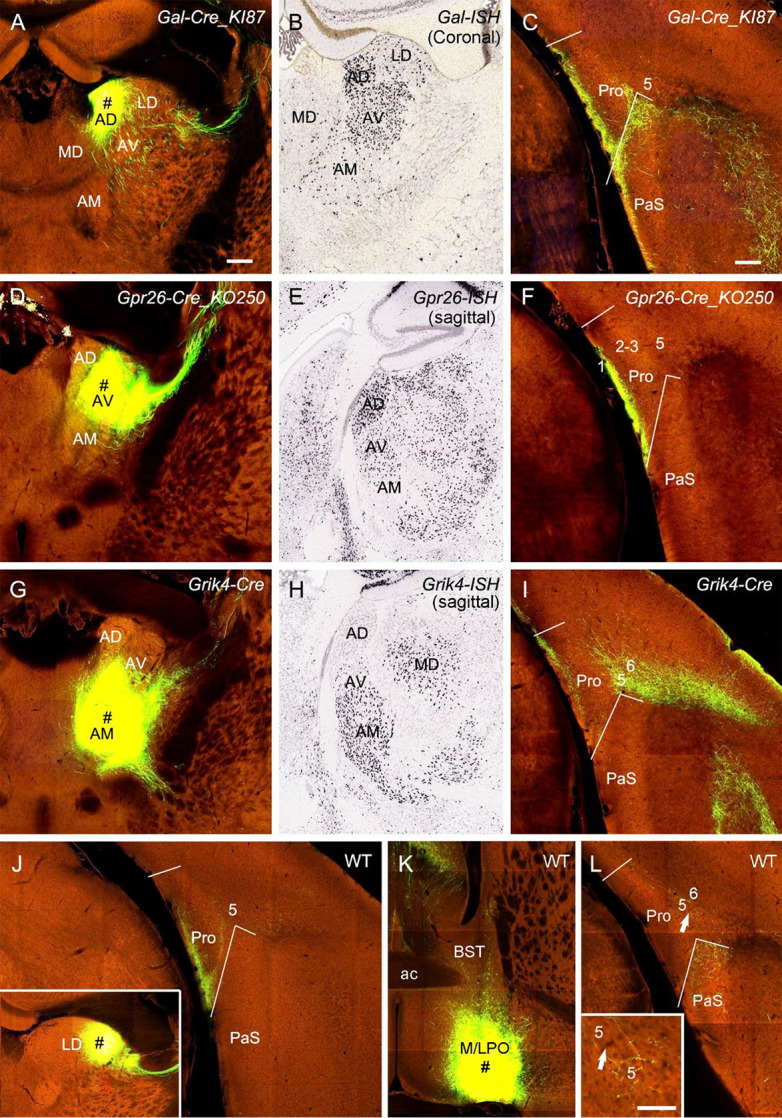
Projections from thalamic and hypothalamic regions to the Pro. **(A–C)** An injection in the anterodorsal nucleus (AD; # in **A**) of a *Gal*-Cre_KI87 mouse, in which strong *Gal*-Cre expression is seen in AD and AV (**B**, for ISH), leads to strong terminal labeling in L1 of the Pro **(C)**. (**D–F)** An injection in the anteroventral nucleus (AV; # in **D**) of a *Gpr26*-Cre_KO250 mouse, in which strong *Gpr26*-Cre expression is seen in AD and AV (**E**, for ISH) and produces strong terminal labeling in L1 of the Pro **(F)**.** (G–I)** An injection in the anteromedial nucleus (AM; # in **G**) and AV of a *Grik4*-Cre mouse, in which strong *Grik4*-Cre expression is seen in AM and AV (**H**, for ISH), results in strong terminal labeling in L1 and L5–6 of the Pro **(I)**. **(J)** Strong terminal labeling in L1 of the Pro after an injection was contained in the laterodorsal nucleus (LD; # in the inset) of a WT mouse.** (K,L)** An injection in the preoptic region of the hypothalamus (M/LPO; # in **K**) of a WT mouse results in weak terminal labeling in L5 of the Pro **(L)**, which is magnified in the inset. The arrows point to the same location. Bar: 350 μm in **(A)** (for all panels); 70 μm in the inset in **(L)**.

ATN were mentioned to project to the Pro (Ding, 2013). ATN mainly includes anterodorsal (AD), anteroventral (AV), and anteromedial (AM) nuclei. To examine the laminar distribution of the projections from these nuclei to the Pro, we selected and analyzed three types of Cre-driver mice: *Gal*-Cre_KI87 (two cases), *Gpr26*-Cre_KO250 (one case), and *Grik4*-Cre (one case) for targeting AD, AV, and AM, respectively (Figures 10A,D,G). *Gal*-Cre and *Gpr26*-Cre are mainly expressed in AD and AV with much fewer in AM while* Grik4*-Cre is mainly expressed in AV and AM with no in AD (Figures 10B,E,H). The tracing results show that AD, AV, and AM all project strongly to L1 of the Pro (Figures 10C,F,I). Labeled terminals in L1 of the Pro in the AM injection case could originate from AV rather than from AM since the AM injection also involved in AV (Figure 10G). AM projects strongly to L5 and less strongly to L6 of the Pro (Figure 10I). Additionally, in four cases with injections in the laterodorsal nucleus [LD; two *Prkcd*-GluCla-CFP-IRES-Cre (Cre in LD) and two WT mice], strongly labeled terminals were seen in L1 of the Pro. An example from one WT mouse is shown in Figure 10J. As for the hypothalamus, the injection in medial and lateral preoptic regions (MPO-LPO) of a WT mouse results in weakly labeled terminals in L5 of the Pro with stronger labeling in adjoining PaS (Figures 10K,L). Scattered terminals were also seen in the Pro after an injection in the dorsal portion (lateral to paraventricular nucleus) of the anterior hypothalamic region in a WT mouse (not shown).

**Figure 11:**
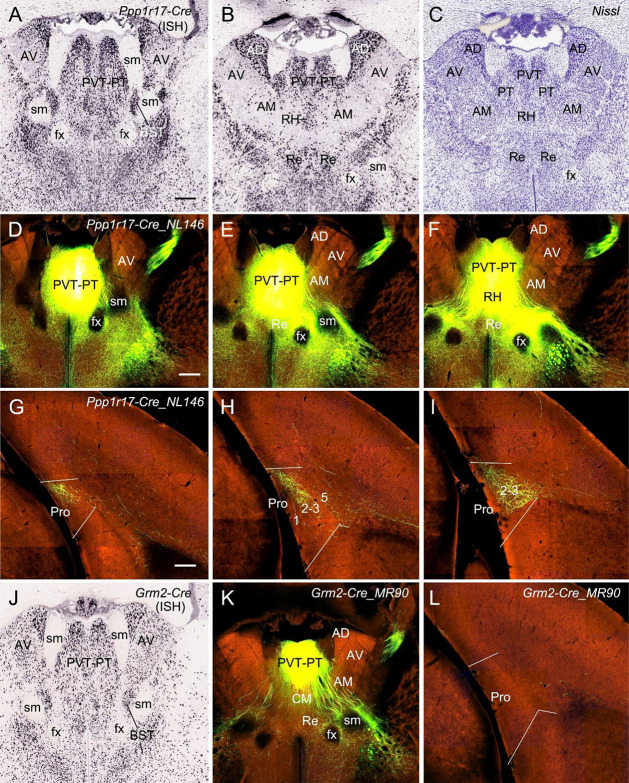
Projections from anterior midline thalamic region to the Pro. **(A–B)** Two sequential sections of the anterior midline thalamic region (ATh) showing *Ppp1r17*-Cre expression in paraventricular-parataenial nuclei (PVT-PT) and adjoining RH, AD, and AM. **(C)** A Nissl-stained section adjacent to **(B)** showing the architecture of the PVT-PT and adjoining regions. **(D–I)** One large injection in the PVT-PT and the adjoining ATh structures **(D–F)** of a *Ppp1r17*-Cre_NL146 mouse leads to strong terminal labeling in L2–3 of the Pro with much fewer in deep L1 **(G–I)**. Panels **(D–F)** are three serial sections of the ATh, and **(G–I)** are three sequential sections of the prostriata region. **(J)** One section at the level **(A)** showing *Grm2*-Cre expression in the PVT-PT and adjoining regions. **(K,L)** One smaller injection in the PVT-PT **(K)** of a *Grm2*-Cre_MR90 mouse produces no terminal labeling in the Pro **(L)**. Bar: 330 μm in **(A)** (for **A–C** and **J**); 350 μm in **(D)** (for **D–F, K**); and 200 μm in **(G)** (for **G–I** and **L**).

Finally, in one *Ppp1r17*-Cre_NL146 mouse, in which strong *Ppp1r17-*Cre expression is seen in paraventricular (PVT) and parataenial (PT) nuclei of the thalamus (PVT-PT) as well as adjoining regions such as rhomboid nucleus (RH), central medial nucleus (CM), a part of bed nucleus of stria terminalis (BST), and medial part of AM [together loosely refer to as the anterior midline thalamic region (ATh); Figures 11A–C], the injection involved in these regions (Figures 11D–F) yields strong terminal labeling in L2–3 (the dorsal part) of the Pro with much less in deep L1 along the rostral-caudal axis (Figures 11G–I). No terminal labeling was observed in adjoining RS, PrS, and PaS. However, in other eight cases (three WT and five different Cre-driver mice) in which the injections were placed in PVT and/or PT or adjoining regions of the ATh, no or little terminal labeling was detected in the Pro. The five Cre-driver lines are *Lypd6*-Cre_KL156 (Cre expression and the injection in PVT, RH, and CM), *Efr3a*-Cre_NO108 (Cre and injection in PVT and MD), *Cck*-IRES-Cre (Cre and injection in PT, BST, AM, CM, and Re), Slc17a6-IRES-Cre (Cre and injection in PT, BST, and AM), and Grm2-Cre_MR90 (Cre and injection in PVT-PT; see Figure 11J). The tracing result from the *Grm2*-Cre mouse is shown in Figures 11K,L. These findings together do not reveal a clear image about the structures of origin of the projections to L2–3 of the Pro although it is clear the cells of origin are a subset of *Ppp1r17*-expressing neurons in the ATh. Further investigation is needed to find out specific structures that originate these projections to the Pro.

## Discussion

**Figure 12:**
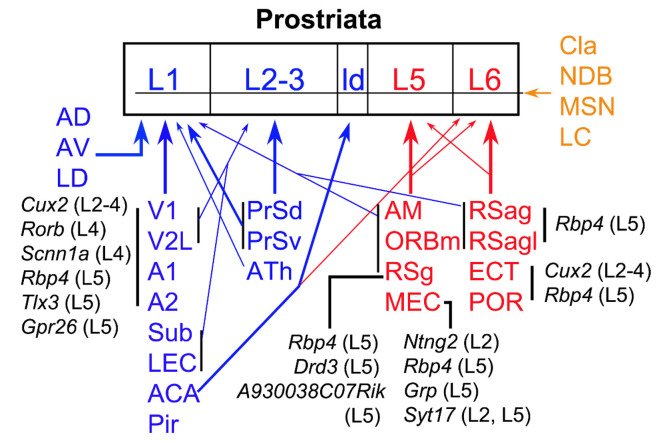
Summary of afferent projections of the prostriata. The regions sending afferents to the Pro are coded blue (to superficial layers), red (to deep layers), and brown (to all layers). Thick and thin arrows indicate moderate/strong and weaker projections, respectively. The cells of origin and their locations/layers in major cortical regions are in black. The cell types that originate the projections to the Pro in specific cortical regions are represented by the genes they express while their laminar locations are indicated in the brackets. For example, *Rbp4* (L5) means *Rbp4* expressing neurons in layer 5; it also indicates that the Cre-driver line *Rbp4*-Cre_KL100 was used in this study to determine the projections (for details see text). ATh, anterior midline thalamic region.

In this study, we investigate brain-wide projections to the Pro using both WT and different Cre-driver mice. Cell type-, region-, and layer-specific projections are found to different layers of the Pro (Figure 12). In general, L1 of the Pro receives its inputs from visual and auditory sensory cortices and many limbic structures while L2–3 mainly receives its afferents from PrS and ATh. L5 of the Pro is the main target of MEC, RSg ORBm, and AM while L6 is mainly targeted by RSag, RSagl, ECT, and POR (see Figure 12). The visual and auditory cortices likely deliver motion-related information about the objects in peripheral environment while different limbic structures such as MEC, PrS, Sub, RS, and ATN likely process spatial and navigation information. We also find that Cla, important to attention and related behavior, innervate all layers of the Pro.

### Cell Type-Specific Projections to the Pro

To gain insight about the cells of origin of the projections to the Pro, tracing data from many cell type-specific Cre-driver mouse lines were examined in this study. For targeting specific neurons in neocortical regions (A1, A2, V2L, POR, and ECT), injections were placed in *Cux2*-IRES-Cre (for subsets of L2–4 neurons), *Rbp4*-Cre_KL100 and/or *Tlx3*-Cre_PL56 (for subsets of L5 neurons), and *Ntsr1*-Cre_GN220 and/or *Syt6*-Cre_KI148 (for subsets of L6 neurons) mice. The results from this Cre-dependent tracing indicate that subsets of L2–4 and L5 neurons but not L6 neurons originate the projections to the Pro (Figure 12). We also find terminal labeling in the Pro after A1 injection in one* Rorb*-IRES2-Cre mouse and one *Scnn1a*-Tg3-Cre (Cre expression in subsets of L4 neurons) mouse. However, no labeling is seen in the Pro after A1 injection in one *Rasgrf2*-T2A-dCre (Cre in subsets of L2–3 neurons) mouse. These results together suggest that L4 of the A1 originate some projections to the Pro although the possibility of some projections from L2–3 to the Pro could not be totally ruled out.

In contrast to the neocortex, the projections from MEC to the Pro appear to originate mainly from L2 and L5 since the terminal labeling is only seen in *Ntng2*-IRES2-Cre (Cre in subsets of L2 neurons), *Rbp4*-Cre_KL100 and *Grp*-Cre_KH288 (Cre in subsets of L5 neurons), and *Syt17*-Cre_NO14 (Cre in subsets of L2 and L5 neurons) mice.

Pro inputs from the RS are originated from subsets of L5 neurons since terminal labeling in the Pro is only seen in *Rbp4*-Cre_KL100, *A930038C07Rik*-Tg1-Cre, and *Drd3-Cre-KI196* (Cre in subsets of L5 neurons in each case) mice. However, no terminal labeling was detected in the Pro in other L5 Cre-driver lines such as Etv1-CreERT2, Adcyap1–2A-Cre, and Efr3a-Cre. This difference is likely the reflection of the existence of different cell types in L5 of the RS. Some subsets of L5 neurons may target Pro while other subsets may innervate other regions (but not the Pro). However, other possibilities may also exist. For example, the injections are not large enough to produce terminal labeling and/or Cre expression is not strong enough to yield labeling. Cell type-specific projections would also exist in other cortical regions and layers.

### Direct Projections From Primary Sensory Cortices to the Pro

Generally, sensory information from peripherals to primary sensory cortices is further directed to higher order of the sensory cortices (association cortices) after some degree of processing and integration in the primary sensory cortices. After further processing and integration, the output information of the association cortices is then forwarded to related limbic cortices, which finally send their outputs to the structures responsible for adaptive actions including related cognitive and behavior changes or modulations (Mesulam, 1998; Harris et al., 2019; but see Rockland, 2015). As a limbic cortex, the Pro does receive inputs from visual and auditory association cortices (e.g., V2L and A2), as revealed in this study. What is unique to the Pro is that it receives direct projections from primary visual (Lu et al., 2020) and primary auditory (this study) cortices. These direct pathways could enable the Pro fast access to the status about moving objects and sounds in peripheral environment. This is consistent with the functional roles of the Pro in fast analysis of moving objects in the far peripheral visual field (Yu et al., 2012; Mikellidou et al., 2017; Tamietto and Leopold, 2018) although specific roles of the Pro in processing moving sounds are currently not clear. When a threatening moving object and/or its sound appears in the environment, the Pro could quickly process the information and initiate adaptive responses *via* its efferent projections. Our preliminary results in rat indicate that the Pro projects to MEC, RS, ventral lateral geniculate nucleus, and pretectal nuclei (Lu et al., 2020) which directly project to the regions that control and regulate adaptive behaviors such as ACA, caudate–putamen, amygdala, claustrum, hypothalamus, zona incerta, periaqueductal gray, superior colliculus, Edinger–Westphal nucleus, brainstem reticular formation, pontine central gray, parabrachial nucleus, facial nucleus, nucleus prepositus, abducens nucleus, and inferior olive (Moore et al., 2000; Gamlin, 2006; Mitchell et al., 2018). Finally, it should be mentioned that the Pro probably does not participate in the processing of somatosensory information since no inputs to the Pro were found from primary and secondary somatosensory cortex and related subcortical regions.

### Laminar Organization of the Inputs to the Pro

Our recent study has revealed strong projections from V1, PrS, and Sub to L1–3 of the Pro (Ding, 2013; Ding et al., 2020; Lu et al., 2020). In this study, we find that the cortical inputs from A1, A2, V2L, LEC, and ACA and the thalamic inputs from AD, AV, and LD all terminate predominantly in L1 with no or much fewer in L2–3 and no in L5–6 (except weak ACA projections to L6; Figure 12). Additional strong projections to L2–3 of the Pro originate from contralateral Pro (Chen et al., 2020) and subsets of *Ppp1r17*-expressing neurons in the ATh (Figure 11), although further studies are needed to confirm the latter. In contrast, the projections from MEC, ORBm, RSg, and AM mainly target L5 and those from the ECT, POR, and RSag mostly innervate L6 of the Pro, although weaker innervation of layer 1 by RSg and RSag is also detected. Overall, the majority of the inputs to the Pro display laminar organization, suggesting that the inputs to the Pro from different sources probably have differential influence on neuronal activity in the Pro. Laminar organization appears to be a common feature of cortical connections since it has been observed in primary sensory, multisensory, and limbic cortices (Foxworthy et al., 2013; D’Souza and Burkhalter, 2017; Rockland, 2019). However, laminar patterns vary in a region-specific manner (Rockland, 2019). The laminar patterns of the Pro inputs described in this study are different from those of other cortical regions including adjoining PrS and RS. This further supports our conclusion that the Pro is a distinct region from PrS and RS (Ding, 2013; Lu et al., 2020). The Pro in rodent was previously treated as part of PrS (Honda et al., 2008; Swanson, 2018) or RS (Haug, 1976; Paxinos and Franklin, 2012), although its existence in nonhuman primate and human brains have been established and confirmed since the later 60 s (Sanides, 1969; Allman and Kaas, 1971; Sousa et al., 1991; Morecraft et al., 2000; Ding et al., 2003, 2016; Rockland, 2012).

### Convergent Projections to Layer 1 of the Pro

In this study, we find limited regions that project to L5 (mainly from AM, RSg, and MEC) and L6 of the Pro (mainly from RSag, POR, and ECT). In contrast, the projections to L1 of the Pro derive from widespread regions including primary and association visual and auditory cortices as well as many limbic structures such as ACA, RS, Sub, PrS, LEC, LD, and ATN. Given that L1 is the major target of feedback projections of many cortical regions (D’Souza and Burkhalter, 2017; Rockland, 2019), these findings suggest that the Pro receives feedback information regarding not only objects and sounds from the environment but also head direction information from the regions containing head direction cells such as PrS, RS, MEC, AD, and LD (Taube, 2007; Giocomo et al., 2014; Preston-Ferrer et al., 2016).

In literature, physiological evidence from single neuron studies of the primate cerebral cortex has suggested that the integration of visual and auditory motion cues may not be mediated by the early visual areas MT and MST and thus the integration likely occurs in higher-level cortical areas (Chaplin et al., 2018). Our finding that both primary and association visual and auditory inputs converge in L1 of the Pro suggests that the Pro is a region for the integration. Moreover, we find that limbic structures such as ATN, LEC, and Sub also heavily innervate L1 of the Pro, suggesting that the Pro is a unique region where primary visual and auditory afferents directly converge with limbic afferents. To our knowledge, this type of convergence was not reported previously. As recently discussed, L1 is an important hub for selectively integrating cortico-cortical and thalamocortical afferents (Chaplin et al., 2018). In L1, the excitation of apical dendrites of deep layer neurons by feedback projections as a way to amplify excitatory inputs to proximal dendrites may be a general mechanism used in cerebral cortex (Phillips, 2017). Specific roles of different inputs to L1 of the Pro remain to be explored in future.

### Diffuse Projections From Cla, LC, and NDB to the Pro

Unlike the laminar innervation of the Pro inputs mentioned above, moderate to strong inputs from the Cla were observed to innervate all layers of the Pro with more heavily labeled terminals in L5–6. This finding suggests that Cla may have important impact on neuronal activities of the Pro at different laminar levels. The Cla was suggested to play important roles in detecting novel sensory stimuli, directing attention, setting behavioral states, and salience processing (Brown et al., 2017; Smith et al., 2019). It should also be mentioned that weak diffuse projections from the LC and NDB to L1–6 of the Pro were detected in this study. Both the LC and NDB are implicated in many cognitive processes including sleep-wake, attention, learning, memory, and decision making (Sara, 2009; Mesulam, 2013).

## Data Availability Statement

All datasets presented in this study are included in the article. These data are available online (http://connectivity.brain-map.org) and from the corresponding author upon reasonable request.

## Ethics Statement

The animal study was reviewed and approved by the Institutional Animal Care and Use Committee of the Allen Institute.

## Author Contributions

SD: conceptualization. JH, CC, and SD: investigation and analysis. JH and SD: writing. SD and SC: supervision. All authors have read and approved the submitted manuscript. All authors contributed to the article and approved the submitted version.

## Conflict of Interest

The authors declare that the research was conducted in the absence of any commercial or financial relationships that could be construed as a potential conflict of interest.

## Footnotes

^1^http://connectivity.brain-map.org/

^2^http://help.brain-map.org/display/mouseconnectivity/documentation

^3^http://mouse.brain-map.org/

^4^http://help.brain-map.org/display/mousebrain/documentation

## Abbreviations

A1: primary auditory cortex
A2: secondary auditory cortex
ACA: anterior cingulate area
AD: anterodorsal nucleus of thalamus
AM: anteromedial nucleus of thalamus
ATN: anterior thalamic nuclei
AV: anteroventral nucleus of thalamus
BL: basolateral amygdaloid nucleus
BST: bed nucleus of stria terminalis
Ca: caudate
CE: central amygdaloid nucleus
CM: central medial nucleus
Cla: claustrum
DLG: dorsal lateral geniculate nucleus
EC: entorhinal cortex
ECT: ectorhinal cortex
fx: fornix
La: lateral amygdaloid nucleus
LC: locus ceruleus
ld: lamina dissecans
LD: lateral dorsal nucleus of thalamus
LEC: lateral entorhinal cortex
LPO: lateral preoptic area
LSN: lateral septal nucleus
MD: mediodorsal thalamic nucleus
MEC: medial entorhinal cortex
MG: medial geniculate nucleus
MPO: medial preoptic area
MSN: medial septal nucleus
NDB: nucleus of diagonal band
ORBm: medial orbitofrontal cortex
PaS: parasubiculum
Pir: piriform cortex
POR: postrhinal cortex
PrS: presubiculum
PrSd, PrSv: dorsal and ventral presubiculum
PT: parataenial nucleus
PTN: pretectal nucleus
PVT: paraventricular thalamic nucleus
Re: reuniens nucleus
RH: rhomboid nucleus
RS: retrosplenial cortex
RSg: granular retrosplenial cortex
RSag: agranular retrosplenial cortex
RSagl: lateral agranular retrosplenial cortex
S1: primary somatosensory cortex
SC: superior colliculus
scp: superior cerebellar peduncle
sm: stria medullaris of thalamus
Sub: subiculum
V2L: lateral secondary visual cortex
V2M: medial secondary visual cortex
VISal: secondary visual cortex, anterolateral subfield
VISl: secondary visual cortex, lateral subfield
VISpl: secondary visual cortex, posterolateral subfield
wm: white matter.
